# The genome sequence of the Atlantic Strawberry Cockle, *Americardia media* (Linnaeus, 1758) (Cardiida: Cardiidae)

**DOI:** 10.12688/wellcomeopenres.25082.1

**Published:** 2025-11-06

**Authors:** Ruiqi Li, Jingchun Li, Wellcome Sanger Institute Tree of Life Management, Samples and Laboratory team, Wellcome Sanger Institute Scientific Operations: Sequencing Operations, Wellcome Sanger Institute Tree of Life Core Informatics team, Tree of Life Core Informatics collective, Mark L. Blaxter

**Affiliations:** 1 Department of Ecology and Evolutionary Biology, University of Colorado Boulder, Boulder, Colorado, USA; 2 Museum of Natural History, University of Colorado Boulder, Boulder, Colorado, USA; 3 Department of Biological Sciences, University of Southern California, Los Angeles, California, USA; 4 Tree of Life, Wellcome Sanger Institute, Hinxton, England, UK

**Keywords:** Americardia media; Atlantic Strawberry Cockle; genome sequence; chromosomal; Cardiida

## Abstract

We present a genome assembly from an individual *Americardia media* (Atlantic Strawberry Cockle; Mollusca; Bivalvia; Cardiida; Cardiidae). The assembly contains two haplotypes with total lengths of 1 299.87 megabases and 1 284.99 megabases. Most of haplotype 1 (99.42%) is scaffolded into 19 chromosomal pseudomolecules. Haplotype 2 was assembled to scaffold level. The mitochondrial genome has also been assembled, with a length of 47.2 kilobases.

## Species taxonomy

Eukaryota; Opisthokonta; Metazoa; Eumetazoa; Bilateria; Protostomia; Spiralia; Lophotrochozoa; Mollusca; Bivalvia; Autobranchia; Heteroconchia; Euheterodonta; Imparidentia; Neoheterodontei; Cardiida; Cardioidea; Cardiidae; Fraginae; *Americardia*; *Americardia media* (Linnaeus, 1758) (NCBI:txid241156)

## Background

*Americardia media* is a widespread and morphologically variable cockle of the Caribbean and western Atlantic. Its range extends from southern Florida and the Gulf of Mexico southward to Panama, Suriname, and throughout the West Indies ( ter Poorten, 2024). This species inhabits sandy bottoms in shallow, sheltered environments, typically in intertidal and subtidal zones associated with seagrass beds or algal meadows ( ter Poorten, 2024).

*Americardia media* belongs to the subfamily Fraginae (heart cockles), which includes both a non-symbiotic lineage and a symbiotic lineage that maintains photosymbiotic relationships with dinoflagellates of the family Symbiodiniaceae ( Kirkendale, 2009; Li *et al.*, 2020). This diversity makes Fraginae a powerful system for comparative studies on the origins and molecular mechanisms of animal photosymbiosis. While several genomes from photosymbiotic fraginids are already available ( Li *et al.*, 2024a; Li *et al.*, 2024b; Li *et al.*, 2024c), the genome of *A. media* adds an essential non-symbiotic counterpart, enabling investigations into morphological adaptation to photosymbiosis and the divergent evolutionary trajectories of symbiotic versus non-symbiotic bivalves.

**Figure 1.**
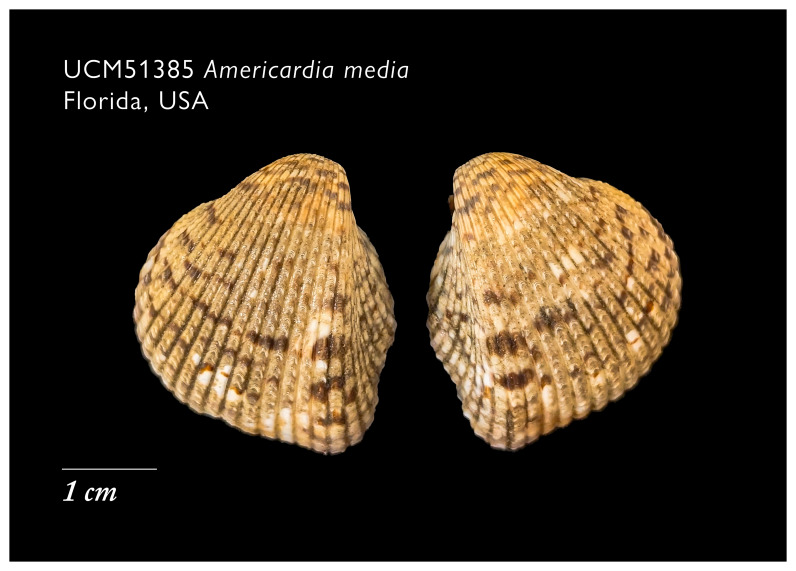
Photograph of the *Americardia media* (xbAmeMedi1) specimen used for genome sequencing.

We present a chromosome-level genome sequence for *Americardia media*, the first genome for the genus *Americardia* and one of 14 genomes available for the family Cardiidae as of September 2025 (data obtained via NCBI datasets, O’Leary *et al.*, 2024). The assembly was produced using the Tree of Life pipeline from a specimen collected in West Palm Beach, Florida, Usa ( Figure 1)

## Methods

### Sample acquisition

The specimen used for genome sequencing was an adult *Americardia media* (specimen ID SAN20002327, ToLID xbAmeMedi1; Figure 1), collected from West Palm Beach, Florida, USA (latitude 26.783, longitude –80.043) on 2022-06-01. The specimen was collected and identified by Ruiqi Li (University of Colorado, Boulder). The voucher id is UCM51385, deposited in the University of Colorado Boulder Museum of Natural History.

### Nucleic acid extraction

Protocols for high molecular weight (HMW) DNA extraction developed at the Wellcome Sanger Institute (WSI) Tree of Life Core Laboratory are available on protocols.io ( Howard *et al.*, 2025). The xbAmeMedi1 sample was weighed and triaged to determine the appropriate extraction protocol. Tissue was homogenised by powermashing using a PowerMasher II tissue disruptor. HMW DNA was extracted using the Automated MagAttract v2 protocol. DNA was sheared into an average fragment size of 12–20 kb following the Megaruptor®3 for LI PacBio protocol. Sheared DNA was purified by automated SPRI (solid-phase reversible immobilisation). The concentration of the sheared and purified DNA was assessed using a Nanodrop spectrophotometer and Qubit Fluorometer using the Qubit dsDNA High Sensitivity Assay kit. Fragment size distribution was evaluated by running the sample on the FemtoPulse system. For this sample, the final post-shearing DNA had a Qubit concentration of 4.4 ng/μL and a yield of 572.00 ng.

### PacBio HiFi library preparation and sequencing

Library preparation and sequencing were performed at the WSI Scientific Operations core. Libraries were prepared using the SMRTbell Prep Kit 3.0 (Pacific Biosciences, California, USA), following the manufacturer’s instructions. The kit includes reagents for end repair/A-tailing, adapter ligation, post-ligation SMRTbell bead clean-up, and nuclease treatment. Size selection and clean-up were performed using diluted AMPure PB beads (Pacific Biosciences). DNA concentration was quantified using a Qubit Fluorometer v4.0 (ThermoFisher Scientific) and the Qubit 1X dsDNA HS assay kit. Final library fragment size was assessed with the Agilent Femto Pulse Automated Pulsed Field CE Instrument (Agilent Technologies) using the gDNA 55 kb BAC analysis kit.

The sample was sequenced on a Revio instrument (Pacific Biosciences). The prepared library was normalised to 2 nM, and 15 μL was used for making complexes. Primers were annealed and polymerases bound to generate circularised complexes, following the manufacturer’s instructions. Complexes were purified using 1.2X SMRTbell beads, then diluted to the Revio loading concentration (200–300 pM) and spiked with a Revio sequencing internal control. The sample was sequenced on a Revio 25M SMRT cell. The SMRT Link software (Pacific Biosciences), a web-based workflow manager, was used to configure and monitor the run and to carry out primary and secondary data analysis.

### Hi-C

***Sample preparation and crosslinking***

The Hi-C sample was prepared from 20–50 mg of frozen tissue of the xbAmeMedi1 sample using the Arima-HiC v2 kit (Arima Genomics). Following the manufacturer’s instructions, tissue was fixed and DNA crosslinked using TC buffer to a final formaldehyde concentration of 2%. The tissue was homogenised using the Diagnocine Power Masher-II. Crosslinked DNA was digested with a restriction enzyme master mix, biotinylated, and ligated. Clean-up was performed with SPRISelect beads before library preparation. DNA concentration was measured with the Qubit Fluorometer (Thermo Fisher Scientific) and Qubit HS Assay Kit. The biotinylation percentage was estimated using the Arima-HiC v2 QC beads.

***Hi-C library preparation and sequencing***

Biotinylated DNA constructs were fragmented using a Covaris E220 sonicator and size selected to 400–600 bp using SPRISelect beads. DNA was enriched with Arima-HiC v2 kit Enrichment beads. End repair, A-tailing, and adapter ligation were carried out with the NEBNext Ultra II DNA Library Prep Kit (New England Biolabs), following a modified protocol where library preparation occurs while DNA remains bound to the Enrichment beads. Library amplification was performed using KAPA HiFi HotStart mix and a custom Unique Dual Index (UDI) barcode set (Integrated DNA Technologies). Depending on sample concentration and biotinylation percentage determined at the crosslinking stage, libraries were amplified with 10 to 16 PCR cycles. Post-PCR clean-up was performed with SPRISelect beads. Libraries were quantified using the AccuClear Ultra High Sensitivity dsDNA Standards Assay Kit (Biotium) and a FLUOstar Omega plate reader (BMG Labtech).

Prior to sequencing, libraries were normalised to 10 ng/μL. Normalised libraries were quantified again and equimolar and/or weighted 2.8 nM pools were created. Pool concentrations were checked using the Agilent 4200 TapeStation (Agilent) with High Sensitivity D500 reagents before sequencing. Sequencing was performed using paired-end 150 bp reads on the Illumina NovaSeq X.

### Genome assembly

Prior to assembly of the PacBio HiFi reads, a database of *k*-mer counts ( *k* = 31) was generated from the filtered reads using FastK. GenomeScope2 ( Ranallo-Benavidez *et al.*, 2020) was used to analyse the *k*-mer frequency distributions, providing estimates of genome size, heterozygosity, and repeat content.

The HiFi reads were assembled using Hifiasm in Hi-C phasing mode ( Cheng *et al.*, 2021; Cheng *et al.*, 2022), producing two haplotypes. Hi-C reads ( Rao *et al.*, 2014) were mapped to the primary contigs using bwa-mem2 ( Vasimuddin *et al.*, 2019). Contigs were further scaffolded with Hi-C data in YaHS ( Zhou *et al.*, 2023), using the --break option for handling potential misassemblies. The scaffolded assemblies were evaluated using Gfastats ( Formenti *et al.*, 2022), BUSCO ( Manni *et al.*, 2021) and MERQURY.FK ( Rhie *et al.*, 2020).

The mitochondrial genome was assembled using MitoHiFi ( Uliano-Silva *et al.*, 2023), which runs MitoFinder ( Allio *et al.*, 2020) and uses these annotations to select the final mitochondrial contig and to ensure the general quality of the sequence. The mitochondrial genome was assembled using OATK ( Zhou *et al.*, 2025).

### Assembly curation

The assembly was decontaminated using the Assembly Screen for Cobionts and Contaminants ( ASCC) pipeline. TreeVal was used to generate the flat files and maps for use in curation. Manual curation was conducted primarily in PretextView and HiGlass ( Kerpedjiev *et al.*, 2018). Scaffolds were visually inspected and corrected as described by Howe *et al*. (2021). Manual corrections included 21 breaks and 44 joins. This reduced the scaffold count by 49.6% and reduced the total assembly length by 1.8%. The curation process is described at https://gitlab.com/wtsi-grit/rapid-curation. PretextSnapshot was used to generate a Hi-C contact map of the final assembly.

### Assembly quality assessment

The Merqury.FK tool ( Rhie *et al.*, 2020) was run in a Singularity container ( Kurtzer *et al.*, 2017) to evaluate *k*-mer completeness and assembly quality for both haplotypes using the *k*-mer databases ( *k* = 31) computed prior to genome assembly. The analysis outputs included assembly QV scores and completeness statistics.

The genome was analysed using the BlobToolKit pipeline, a Nextflow implementation of the earlier Snakemake version ( Challis *et al.*, 2020). The pipeline aligns PacBio reads using minimap2 ( Li, 2018) and SAMtools ( Danecek *et al.*, 2021) to generate coverage tracks. It runs BUSCO ( Manni *et al.*, 2021) using lineages identified from the NCBI Taxonomy ( Schoch *et al.*, 2020). For the three domain-level lineages, BUSCO genes are aligned to the UniProt Reference Proteomes database ( Bateman *et al.*, 2023) using DIAMOND blastp ( Buchfink *et al.*, 2021). The genome is divided into chunks based on the density of BUSCO genes from the closest taxonomic lineage, and each chunk is aligned to the UniProt Reference Proteomes database with DIAMOND blastx. Sequences without hits are chunked using seqtk and aligned to the NT database with blastn ( Altschul *et al.*, 1990). The BlobToolKit suite consolidates all outputs into a blobdir for visualisation. The BlobToolKit pipeline was developed using nf-core tooling ( Ewels *et al.*, 2020) and MultiQC ( Ewels *et al.*, 2016), with containerisation through Docker ( Merkel, 2014) and Singularity ( Kurtzer *et al.*, 2017).

## Genome sequence report

### Sequence data

**Figure 2.**
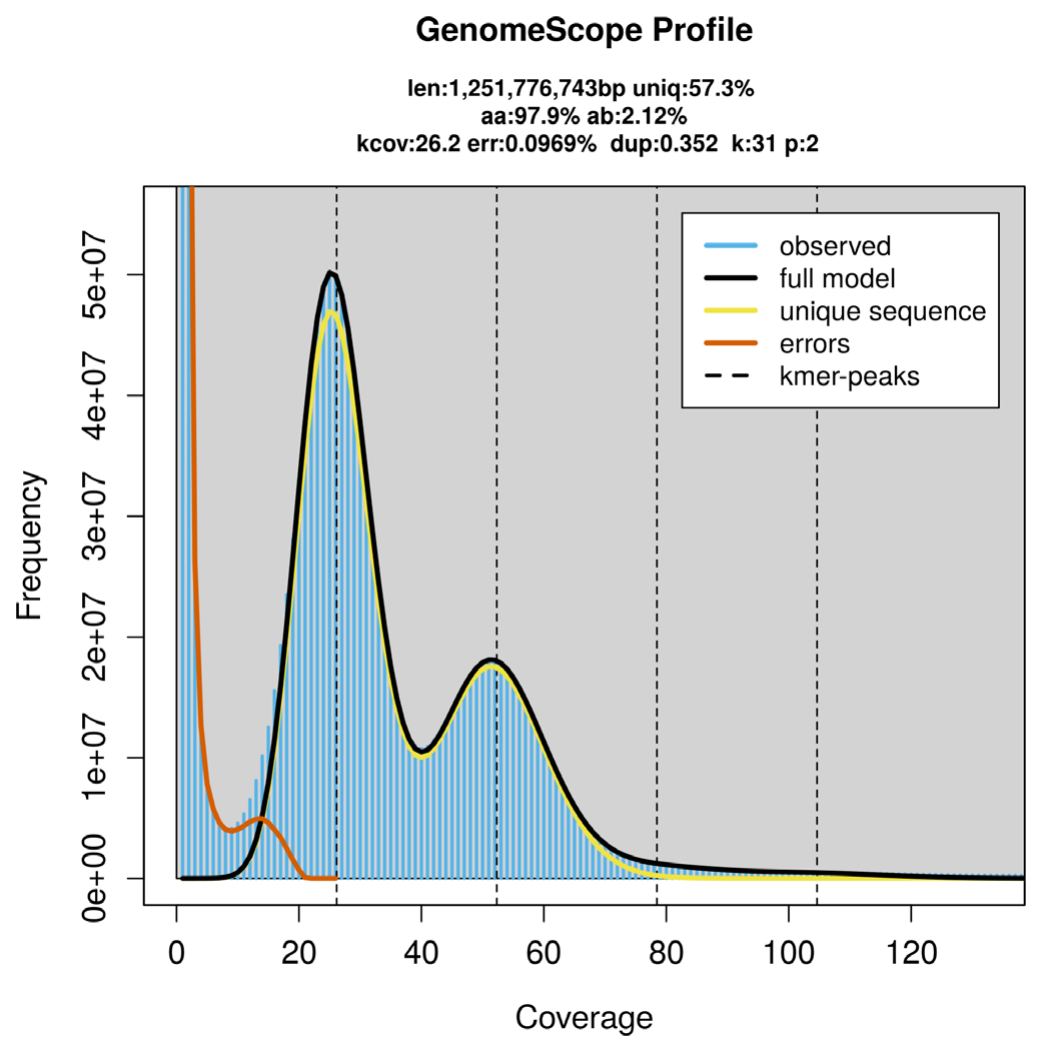
Frequency distribution of *k*-mers generated using GenomeScope2. The plot shows observed and modelled *k*-mer spectra, providing estimates of genome size, heterozygosity, and repeat content based on unassembled sequencing reads.

PacBio sequencing of the *Americardia media* specimen generated 67.68 Gb (gigabases) from 6.66 million reads, which were used to assemble the genome. GenomeScope2.0 analysis estimated the haploid genome size at 1 251.78 Mb, with a heterozygosity of 2.12% and repeat content of 42.88% ( Figure 2). These estimates guided expectations for the assembly. Based on the estimated genome size, the sequencing data provided approximately 52× coverage. Hi-C sequencing produced 112.83 Gb from 747.19 million reads, which were used to scaffold the assembly. Table 1 summarises the specimen and sequencing details.

**Table 1.** Specimen and sequencing data for BioProject PRJEB80831.

Platform	PacBio HiFi	Hi-C
**ToLID**	xbAmeMedi1	xbAmeMedi1
**Specimen ID**	SAN20002327	SAN20002327
**BioSample (source individual)**	SAMEA115336917	SAMEA115336917
**BioSample (tissue)**	SAMEA115336924	SAMEA115336924
**Tissue**	Whole organism	Whole organism
**Instrument**	Revio	Illumina NovaSeq X
**Run accessions**	ERR13762720	ERR13766898
**Read count total**	6.66 million	747.19 million
**Base count total**	67.68 Gb	112.83 Gb

### Assembly statistics

The genome was assembled into two haplotypes using Hi-C phasing. Haplotype 1 was curated to chromosome level, while haplotype 2 was assembled to scaffold level. The final assembly has a total length of 1 299.87 Mb in 267 scaffolds, with 365 gaps, and a scaffold N50 of 67.44 Mb ( Table 2).

**Table 2.** Genome assembly statistics.

**Assembly name**	xbAmeMedi1.hap1.1	xbAmeMedi1.hap2.1
**Assembly accession**	GCA_964300355.1	GCA_964300375.1
**Assembly level**	chromosome	scaffold
**Span (Mb)**	1 299.87	1 284.99
**Number of chromosomes**	19	Scaffold-level
**Number of contigs**	632	507
**Contig N50**	5.6 Mb	5.99 Mb
**Number of scaffolds**	267	174
**Scaffold N50**	67.44 Mb	66.77 Mb
**Longest scaffold length (Mb)**	105.39	-
**Organelles**	Mitochondrion: 47.2 kb	-

**Figure 3.**
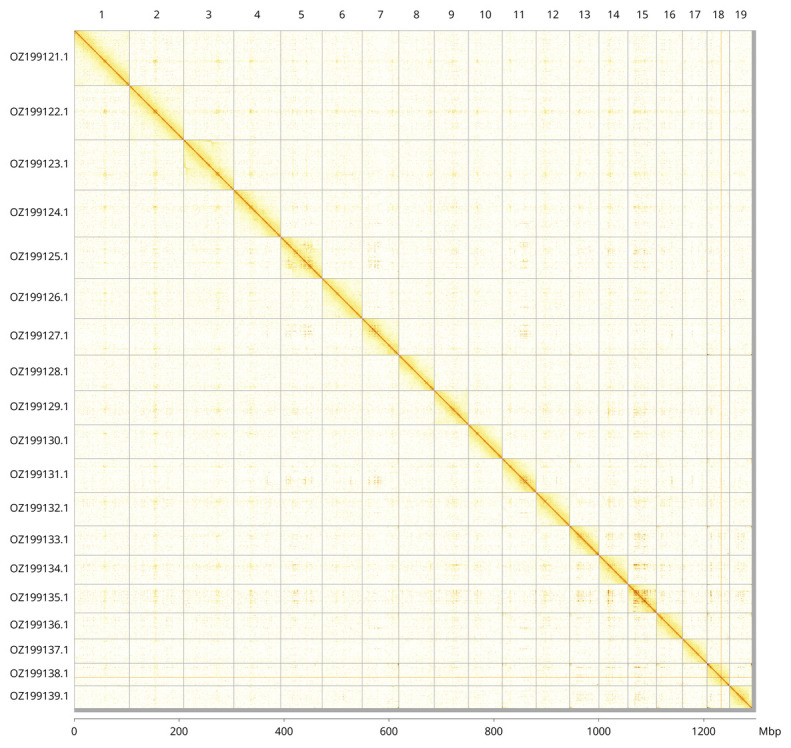
Hi-C contact map of the *Americardia media* genome assembly. Assembled chromosomes are shown in order of size and labelled along the axes, with a megabase scale shown below. The plot was generated using PretextSnapshot.

Most of the assembly sequence (99.42%) was assigned to 19 chromosomal-level scaffolds. These chromosome-level scaffolds, confirmed by Hi-C data, are named according to size ( Figure 3; Table 3). During curation, we observed haplotypic inversions in the following regions: chromosome 3 (4.6–53.7 Mbp) and 5 (21.3–51.2 Mbp).

**Table 3.** Chromosomal pseudomolecules in the haplotype 1 genome assembly of *Americardia media* xbAmeMedi1.

INSDC accession	Molecule	Length (Mb)	GC%
OZ199121.1	1	105.39	36
OZ199122.1	2	103.60	36
OZ199123.1	3	95.37	36
OZ199124.1	4	89.52	36.50
OZ199125.1	5	78.89	36.50
OZ199126.1	6	76.43	36
OZ199127.1	7	69.81	36
OZ199128.1	8	67.44	36
OZ199129.1	9	65.20	36.50
OZ199130.1	10	64.76	36.50
OZ199131.1	11	64.19	36.50
OZ199132.1	12	64.10	36
OZ199133.1	13	55.83	36.50
OZ199134.1	14	55.18	36.50
OZ199135.1	15	54.49	37
OZ199136.1	16	49.71	36.50
OZ199137.1	17	46.47	36.50
OZ199138.1	18	43.02	36.50
OZ199139.1	19	42.96	36.50

The mitochondrial genome was also assembled (length 47.2 kb, OZ199140.1). This sequence is included as a contig in the multifasta file of the genome submission and as a standalone record.

**Figure 4.**
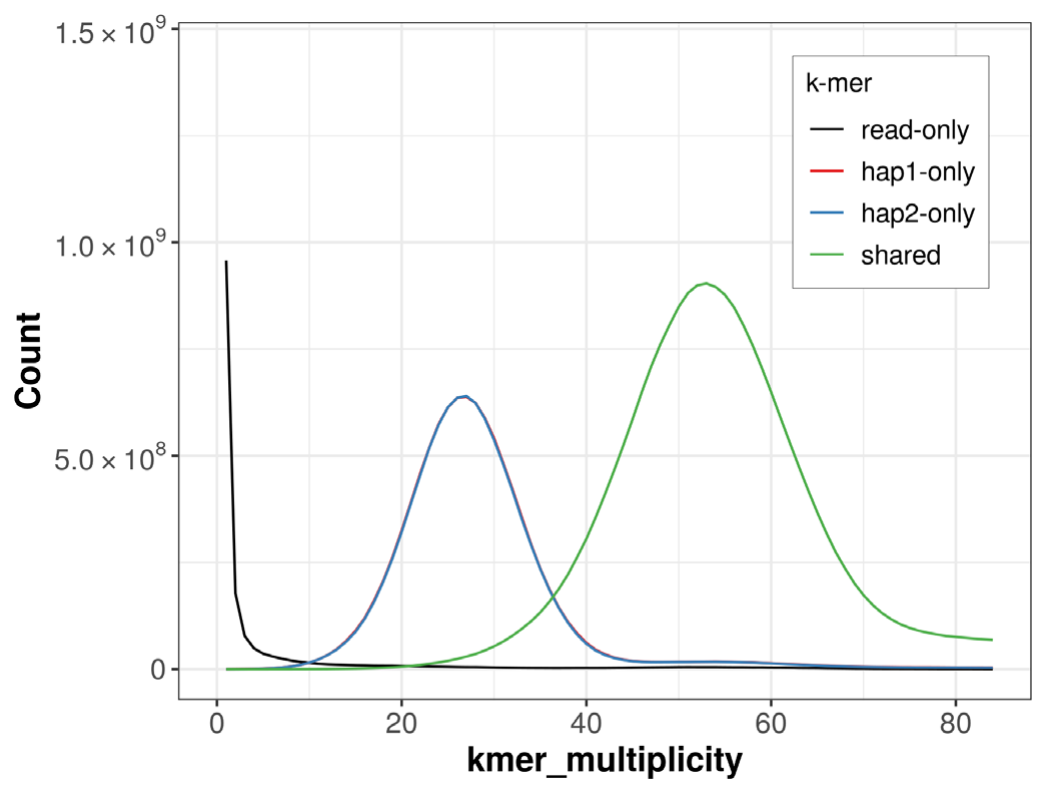
Evaluation of *k*-mer completeness using MerquryFK. This plot illustrates the recovery of *k*-mers from the original read data in the final assemblies. The horizontal axis represents *k*-mer multiplicity, and the vertical axis shows the number of *k*-mers. The black curve represents *k*-mers that appear in the reads but are not assembled. The green curve corresponds to *k*-mers shared by both haplotypes, and the red and blue curves show *k*-mers found only in one of the haplotypes.

For haplotype 1, the estimated QV is 65.9, and for haplotype 2, 66.7. When the two haplotypes are combined, the assembly achieves an estimated QV of 66.3. The *k*-mer completeness is 68.22% for haplotype 1, 67.98% for haplotype 2, and 98.90% for the combined haplotypes ( Figure 4).

**Figure 5.**
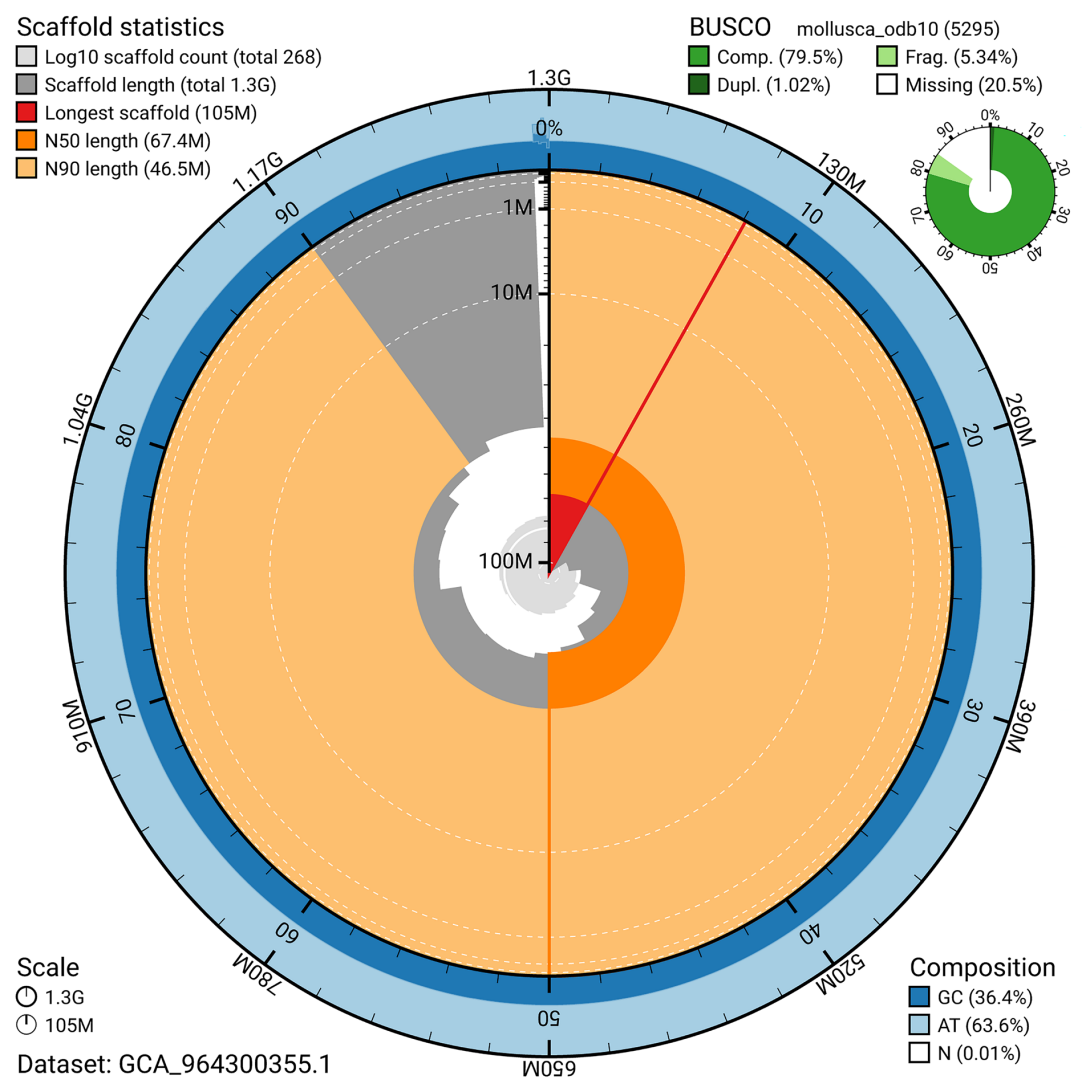
Assembly metrics for xbAmeMedi1.hap1.1. The BlobToolKit snail plot provides an overview of assembly metrics and BUSCO gene completeness. The circumference represents the length of the whole genome sequence, and the main plot is divided into 1 000 bins around the circumference. The outermost blue tracks display the distribution of GC, AT, and N percentages across the bins. Scaffolds are arranged clockwise from longest to shortest and are depicted in dark grey. The longest scaffold is indicated by the red arc, and the deeper orange and pale orange arcs represent the N50 and N90 lengths. A light grey spiral at the centre shows the cumulative scaffold count on a logarithmic scale. A summary of complete, fragmented, duplicated, and missing BUSCO genes in the set is presented at the top right. An interactive version of this figure can be accessed on the BlobToolKit viewer.

**Figure 6.**
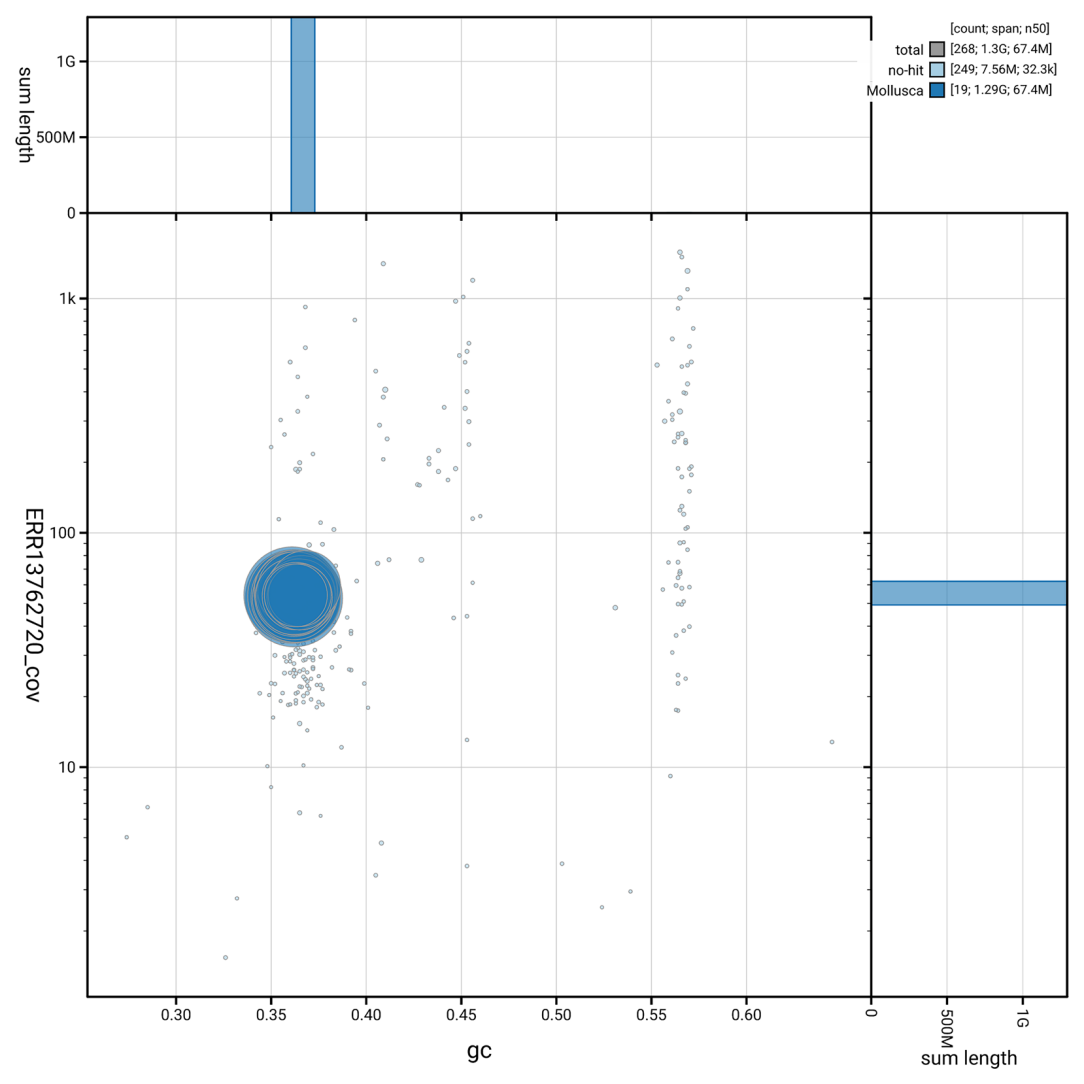
BlobToolKit GC-coverage plot for xbAmeMedi1.hap1.1. Blob plot showing sequence coverage (vertical axis) and GC content (horizontal axis). The circles represent scaffolds, with the size proportional to scaffold length and the colour representing phylum membership. The histograms along the axes display the total length of sequences distributed across different levels of coverage and GC content. An interactive version of this figure is available on the BlobToolKit viewer.

BUSCO analysis using the mollusca_odb10 reference set ( *n* = 5 295) identified 79.5% of the expected gene set (single = 78.5%, duplicated = 1.0%) for haplotype 1. The snail plot in Figure 5 summarises the scaffold length distribution and other assembly statistics for haplotype 1. The blob plot in Figure 6 shows the distribution of scaffolds by GC proportion and coverage for haplotype 1.

Table 4 lists the assembly metric benchmarks adapted from Rhie *et al.* (2021) and the Earth BioGenome Project Report on Assembly Standards September 2024. The EBP metric, calculated for the haplotype 1, is **6.C.Q65**, meeting the recommended reference standard.

**Table 4.** Earth Biogenome Project summary metrics for the *Americardia media* assembly.

Measure	Value	Benchmark
EBP summary (haplotype 1)	6.C.Q65	6.C.Q40
Contig N50 length	5.60 Mb	≥ 1 Mb
Scaffold N50 length	67.44 Mb	= chromosome N50
Consensus quality (QV)	Haplotype 1: 65.9; haplotype 2: 66.7; combined: 66.3	≥ 40
*k*-mer completeness	Haplotype 1: 68.22%; Haplotype 2: 67.98%; combined: 98.90%	≥ 95%
BUSCO	C:79.5% [S:78.5%; D:1.0%]; F:5.3%; M:15.1%; n:5 295	S > 90%; D < 5%
Percentage of assembly assigned to chromosomes	99.42%	≥ 90%

### Wellcome Sanger Institute – Legal and Governance

The materials that have contributed to this genome note have been supplied by a Tree of Life collaborator. The Wellcome Sanger Institute employs a process whereby due diligence is carried out proportionate to the nature of the materials themselves, and the circumstances under which they have been/are to be collected and provided for use. The purpose of this is to address and mitigate any potential legal and/or ethical implications of receipt and use of the materials as part of the research project, and to ensure that in doing so we align with best practice wherever possible.

The overarching areas of consideration are:

- Ethical review of provenance and sourcing of the material
- Legality of collection, transfer and use (national and international)

Each transfer of samples is undertaken according to a Research Collaboration Agreement or Material Transfer Agreement entered into by the Tree of Life collaborator, Genome Research Limited (operating as the Wellcome Sanger Institute) and in some circumstances other Tree of Life collaborators.

## Data Availability

European Nucleotide Archive: Americardia media (Atlantic strawberry cockle). Accession number PRJEB80831. The genome sequence is released openly for reuse. The *Americardia media* genome sequencing initiative is part of the Sanger Institute Tree of Life Programme (PRJEB43745). All raw sequence data and the assembly have been deposited in INSDC databases. The genome will be annotated using available RNA-Seq data and presented through the Ensembl pipeline at the European Bioinformatics Institute. Raw data and assembly accession identifiers are reported in Table 1 and Table 2. Production code used in genome assembly at the WSI Tree of Life is available at https://github.com/sanger-tol. Table 5 lists software versions used in this study.

## Author information

Contributors are listed at the following links:

- Members of the Wellcome Sanger Institute Tree of Life Management, Samples and Laboratory team
- Members of Wellcome Sanger Institute Scientific Operations – Sequencing Operations
- Members of the Wellcome Sanger Institute Tree of Life Core Informatics team
- Members of the Tree of Life Core Informatics collective
