# Supplementary material for: The genome sequence of the Atlantic Strawberry Cockle, Americardia media (Linnaeus, 1758) (Cardiida: Cardiidae)
Source: Wellcome Open Res. 2025 Nov 6;10:630. [Version 1] doi: 10.12688/wellcomeopenres.25082.1 (PMC12756599; doi:10.12688/wellcomeopenres.25082.1)
Supplement: Peer review history

# Reviewer report: Reviewer response for version 1

**DOI:** 10.21956/wellcomeopenres.27651.r139139

**Published:** 2025-12-31

**Authors:** Rebekah L. Rogers

**Affiliations:** 1 UNC Charlotte, Charlotte, North Carolina, USA

This chromosomal assembly for the Atlantic Strawberry Cockle is an impressive genome that overcomes some technical challenges to achieve pseudo-chromosomal assembly of independent haplotypes for a mollusc.  Genome sequencing in mollusca has often produced fragmented genomes and this genome note will be a step forward for the field.  

 All assembly methods and QC appear correct and straightforward to assess.  The data are publicly available.  This manuscript will be a fine resource release for the field of mollusc genomics.

Are sufficient details of methods and materials provided to allow replication by others?

Yes

Is the rationale for creating the dataset(s) clearly described?

Yes

Are the datasets clearly presented in a useable and accessible format?

Yes

Are the protocols appropriate and is the work technically sound?

Yes

Reviewer Expertise:

Evolutionary Genomics

I confirm that I have read this submission and believe that I have an appropriate level of expertise to confirm that it is of an acceptable scientific standard.

# Reviewer report: Reviewer response for version 1

**DOI:** 10.21956/wellcomeopenres.27651.r139138

**Published:** 2025-12-23

**Authors:** Kyle E McElroy

**Affiliations:** 1 Iowa State University, Ames, USA

**Summary**

 The authors report details on the assembly of a chromosome-level genome assembly for a species of bivalve, *Americardia media*, the Atlantic Strawberry Cockle. This species belongs to subfamily within Cardiidae that is notable for including lineages with and without photosymbiotic relationships. With genomes for multiple photosymbiotic species already available, the authors note that this non-photosymbiotic species would serve as an important contrast for comparative genomic studies. PacBio HiFi reads were generated for the initial contig assembly, which was then scaffolded into 19 chromosomal pseudomolecules with Hi-C. The genome assembly for *Americardia media* appears to be of high quality and completeness, as indicated by its contig N50, chromosome resolution, and its BUSCO and QV scores, which are all meet the Earth Biogenome Project standards. The genome was also phased into two haplotypes. The methods and results are clear and sufficiently detailed. I only have a few minor comments.

**Minor comments**

 In the Background, I would like to see some description of the Earth Biogenome Project goals and how this resource fits in.

 Given the Earth Biogenome Project benchmarks of a BUSCO completion score of >90%, the authors might want to report the metazoan db results in Table 4, which are much higher than the molluscan db results that tend to be low for even high quality assemblies.

 Regarding the Hi-C methods, does the kit rely on a restriction enzyme with site-specificity or was it non-specific like DNAse I? Otherwise, I appreciate the amount of detail in the Hi-C preparations.

 Out of general curiosity, was RNA-seq not generated? This line: “The genome will be annotated using available RNA-Seq data …” implies that some already exists, is that the case?

 There are two mentions of mitochondrial genome assembly (from the same paragraph) that seem to be at odds with one another. Also, the mitochondrial contig should be in Table 3.

- “The mitochondrial genome was assembled using MitoHiFi (Uliano-Silva *et al.*, 2023), which …”
- “The mitochondrial genome was assembled using OATK (Zhou *et al.*, 2025).”

Are sufficient details of methods and materials provided to allow replication by others?

Yes

Is the rationale for creating the dataset(s) clearly described?

Yes

Are the datasets clearly presented in a useable and accessible format?

Yes

Are the protocols appropriate and is the work technically sound?

Yes

Reviewer Expertise:

Evolutionary biology, genomics, genome evolution, bivalves, gene-family evolution, opsins, asexual reproduction, transposable elements

I confirm that I have read this submission and believe that I have an appropriate level of expertise to confirm that it is of an acceptable scientific standard.
